# The Link between Dengue Incidence and El Niño Southern Oscillation

**DOI:** 10.1371/journal.pmed.1000185

**Published:** 2009-11-17

**Authors:** Pejman Rohani

**Affiliations:** 1 Department of Ecology & Evolutionary Biology, University of Michigan, Ann Arbor, Michigan, United States of America; 2 Center for the Study of Complex Systems, University of Michigan, Ann Arbor, Michigan, United States of America

## Abstract

Pejman Rohani discusses a new study that examined the dynamic relationship between climate variables and dengue incidence in Thailand, Mexico, and Puerto Rico.

**Linked Research Article**

This Perspective discusses the following new study published in *PLoS Medicine*:

Johansson MA, Cummings DAT, Glass GE (2009) Multi-Year Variability and Dengue: El Niño Southern Oscillation, Weather, and Dengue Incidence in Puerto Rico, Mexico, and Thailand. PLoS Med 6(11): e1000168. doi:10.1371/journal.pmed.1000168

*“Prediction is very difficult, especially about the future.”*

*Niels Bohr*

The current H1N1 influenza pandemic has highlighted the practical usefulness of knowing ahead of time about an impending large outbreak. Specifically, as the flu season in the northern hemisphere gets into full swing, public health decision makers have been using information gleaned from epidemics in Mexico and the US earlier this spring to prepare proactive mitigation and control strategies.

The World Health Organization (WHO) has recently focused considerable attention on the development of early warning systems [1], whereby reliable predictors of epidemics may be usefully employed for preparedness. For the most part, this effort has centered on examining whether climatological variables can be studied for epidemic prediction purposes. There are known mechanisms linking environmental conditions, such as rainfall and temperature, with important determinants of disease transmission [2]. These mechanisms may affect both the causative agents and any associated vector species. For example, temperature determines both the developmental rate of the malaria parasite *Plasmodium falciparum* [3] and the persistence of avian influenza viruses in aquatic environments [4], with implications for transmission dynamics [5].

The most obvious—and well-studied—impact of climatic variables is on disease vectors. It is well established that the abundance and distribution of insect vectors is well explained by patterns of temperature, humidity, and rainfall. But these factors also affect vectors at a much smaller scale, by modulating metabolic activity, egg production, and, critically, feeding behavior [6].

In addition to the existence of plausible climatological determinants of epidemic risk, the implementation and reliability of early warning systems requires empirical proof of concept. This requirement has led a number of scientists to examine the association between annual and multi-annual climate variation and the incidence and dynamics of infectious diseases [2],[7], especially malaria [8],[9], with an eye to making predictions about the consequences of climate change [10],[11].

## Insights from a New Analysis

In a new study by Johansson and colleagues published in this issue of *PLoS Medicine* [12], the authors carry out statistical time-series analyses to examine the dynamic relationship between climate variables and the incidence of dengue in Thailand, Mexico, and Puerto Rico. They find no systematic association between multi-annual dengue outbreaks and El Niño Southern Oscillation.

Dengue fever, also known as breakbone fever, is caused by four closely related viral serotypes, members of the Flaviviridae. In recent decades, there has been an alarming increase in the geographical range and severity of dengue epidemics. The infection is now considered endemic in more than 100 countries. The WHO estimates that almost half the world's population is currently at risk, with perhaps as many as 50 million annual cases of dengue infection globally [13]. Clearly, understanding the underlying causes of these trends in dengue incidence and spread has become a research priority.

Dengue transmission occurs via an insect vector, predominantly *Aedes aegytpi* but also *Ae. albopictus*. Environmental parameters, especially temperature and precipitation, affect the demography and behavior of these vectors, making dengue an obvious candidate for researching the impact of climate on disease. Johansson and colleagues show that dengue incidence is very strongly seasonal, in all three countries they studied. This seasonality is the footprint of local weather variables, which also vary seasonally, and their impact on the demography of the mosquito vectors and transmission dynamics [14]. Additionally, however, there are longer multiennial or multiyear variations in dengue incidence. These variations may involve other climate factors, such as the El Niño Southern Oscillation (ENSO, a periodic variation in the atmospheric conditions and ocean surface temperatures of the tropical Pacific). The challenge is to tease apart the relative impacts of mechanisms that are intrinsic to the host–pathogen interaction (such as human demography [15] and immune-mediated serotype interactions [16]) and climatic drivers such as ENSO.

Johansson and colleagues use wavelet coherence analysis [17] to study climate data, including ENSO and local weather variables, and examine the statistical association with dengue incidence. The significance of any association is tested by comparison with bootstrapped-simulated data that have similar statistical properties as their data (specifically, the same autocorrelation structure, which describes the “relatedness” of successive points in the time series). The authors observe very notable geographical variation in the observed impact of ENSO on dengue incidence. In Mexico, they found no association, while in Thailand, the association was not statistically significant. In contrast, in Puerto Rico, a statistically significant association was found between 1995 and 2002. Sensibly, the authors scrutinized these results further by examining the impact of ENSO on local precipitation levels and the subsequent observed dengue incidence. On the basis of biological implausibility (for example, the observation that precipitation increased after dengue outbreaks), the authors argue that their findings from the Puerto Rico data need to be viewed with caution.

## Public Health Implications

The absence of a predictable consequence of ENSO for dengue transmission is an important piece of information for the development of early warning systems. However, the authors are very careful in interpreting their findings and suggest that given the known mechanistic environmental drivers of vector biology, it remains formally possible that ENSO might play a role in dengue transmission. In this light, their findings may be explained along two distinct lines. They may reflect inadequacies in the time-series data (ideally, they would span a longer period and contain higher spatial resolution), or that intrinsic processes such as human birth rates or serotype interactions via immunological mechanisms simply swamp any signatures of the impact of ENSO. For these reasons, this careful work by Johansson et al. is both scientifically interesting and very timely.
